# Anti-Inflammatory Property of 5-Demethylnobiletin (5-Hydroxy-6, 7, 8, 3′, 4′-pentamethoxyflavone) and Its Metabolites in Lipopolysaccharide (LPS)-Induced RAW 264.7 Cells

**DOI:** 10.3390/biology11121820

**Published:** 2022-12-14

**Authors:** Shanshan Guo, Xian Wu, Jinkai Zheng, Mingyue Song, Ping Dong, Hang Xiao

**Affiliations:** 1 Department of Food Science and Nutrition, University of Jinan, Jinan 250022, China; 2 Department of Food Science, University of Massachusetts Amherst, Amherst, MA 01003, USA; 3 Department of Kinesiology, Nutrition, and Health, Miami University, Oxford, OH 45056, USA; 4 Institute of Agro-Products Processing Science and Technology, Chinese Academy of Agricultural Sciences, Beijing 100193, China; 5 College of Food Science, South China Agricultural University, Guangzhou 510642, China; 6 College of Food Science and Engineering, Ocean University of China, Qingdao 266003, China

**Keywords:** 5-demethylnobiletin, metabolites, anti-inflammation, RAW 264.7 macrophages

## Abstract

**Simple Summary:**

5-Demethylnobiletin (5-hydroxy-6, 7, 8, 3′, 4′-pentamethoxyflavone, 5DN) is a bioactive polymethoxyflavone mainly found in citrus plants. To our knowledge, the present study is the first report on the inhibitory mechanism of 5DN and its metabolites on LPS-induced inflammation in RAW 264.7 macrophage cells. Importantly, 5DN’s metabolites showed more potent activities than the parent compound. The possible relationship between the structural properties of 5DN and its metabolites and their anti-inflammatory activity deserves further investigation.

**Abstract:**

Hydroxylated polymethoxyflavones (PMFs) are a unique class of flavonoid compounds mainly found in citrus plants. We investigated the anti-inflammatory effects of one major 5-hydroxy PMF, namely 5-demethylnobiletin (5DN) and its metabolites 5, 3′-didemethylnobiletin (M1), 5, 4′-didemethylnobiletin (M2), and 5, 3′, 4′-tridemethylnobiletin (M3) in lipopolysaccharide (LPS)-stimulated RAW 264.7 macrophage cells. The results showed that M2 and M3 produced stronger inhibitory effects on the production of nitric oxide (NO) than their parent compound at non-cytotoxic concentrations. Western blotting and real-time PCR analyses demonstrated that M2 and M3 significantly decreased iNOS and COX-2 gene expression. The results also showed that M1 and M3 induced heme oxygenase-1(HO-1) gene expression. Overall, our results demonstrated that metabolites of 5DN significantly inhibited LPS-induced inflammation in RAW 264.7 macrophage cells and generally possessed more potent anti-inflammatory activity than the parent compound, 5DN.

## 1. Introduction

Controlling inflammation has been recognized as one important strategy for the prevention and treatment of cancer [1]. Polymethoxylflavonoids (PMFs) are a kind of natural flavonoids with two or more methoxylates in the parent nucleus of 2-phenyltryptophenone. PMFs are widely found in citrus plants, especially in the peel of sweet oranges (*Citrus sinensis*) and citrus reticulate (*Citrus reticulate*) [2]. PMFs have a wide range of biological activities, including anti-inflammatory [3,4,5], anti-carcinogenic [4,5,6,7,8], and anti-atherosclerosis [9,10]. Permethoxylated PMFs, such as nobiletin, 3, 5, 6, 7, 8, 3′, 4′-heptamethoxylflavone (HMF), and tangeretin, are generally well studied. In recent decades, a unique class of hydroxylated PMFs was isolated from long-placed citrus peel extracts, which could be formed by automatic hydrolysis of their holomethoxylated parent compound during prolonged placement [2,11,12]. 5-Demethylnobiletin (5-hydroxy-6, 7, 8, 3’, 4’-pentamethoxyflavone, 5DN) is a hydroxylated PMF that can be synthesized from nobiletin. The 5-position methoxy group of ring A in the chemical structure of nobiletin is hydroxylated.

It was found that 5DN inhibited the production and gene expression of matrix metalloproteinase 9 [13], suppressed non-enzymatic lipid peroxidation [14], and removed superoxide free radicals in non-enzymatic systems [15]. In TPA-induced otitis in mice, 5DN reduced edema, inflammatory cell infiltration, and tissue damage [16]. 5DN also inhibited the growth of a variety of cancer cells, such as colon cancer [17], cervical cancer [18], and leukemia [11] cells. Studies have shown that some hydroxylated PMFs had more potent biological activity than their parent PMFs [5,19]. For example, it was found that 5-hydroxy-PMFs exerted stronger anti-colon cancer activity than their parent PMFs [17].

We reported previously that 5, 3′-didemethylnobiletin (5, 3′-dihydroxy-6, 7, 8, 4′-tetramethoxylflavone, M1), 5, 4′-didemethylnobiletin (5, 4′-dihydroxy-6, 7, 8, 3′-tetramethoxylflavone, M2), and 5, 3′, 4′-tridemethylnobiletin (5, 3′, 4′-trihydroxy-6, 7, 8-trimethoxylflavone, M3) were the three main metabolites of 5DN in mouse urine [20]. It has been reported that M2 suppressed NO production in activated macrophages [21]. However, the comparative inhibitory mechanisms of 5DN and its three metabolites on inflammation have not been reported. In this study, we mainly focused on the inhibitory mechanisms of 5DN and its metabolites M1, M2, and M3 on LPS-induced inflammation in RAW 264.7 macrophage cells.

## 2. Materials and Methods

### 2.1. Chemicals

Nobiletin (purity ≥ 98%) was purchased from Quality Phytochemicals LLC (Edison, NJ, USA). Organic solvents and all reagents for isolation, chemical synthesis, and HPLC analysis were purchased from Fisher Scientific (Fairlawn, NJ, USA). 5DN (yield 94%) was directly obtained as a pale-yellow crystal solid through acid hydrolysis (reflux in 3 M HCl for 96 h) from nobiletin [20]. M1, M2, and M3 were chemically synthesized, as reported previously [20].

### 2.2. Cell Culture

RAW 264.7 cells were purchased from the American Type Culture Collection (ATCC, Rockville, MD, USA) and were cultured in RPMI-1640 medium containing 10% heat-inactivated calf serum, 100 U/mL penicillin and 0.1 mg/mL streptomycin, and incubated in an incubator at 37 °C and 5% CO_2_. All the cells used in the experiment were from 3 to 30 generations. DMSO was used as the solvent of 5DN and its metabolites in the experiment, and its final concentration was ≤0.1% (*v*/*v*).

### 2.3. Cell Viability Assay

Cell viability was determined as previously described [19]. RAW 264.7 cells at the logarithmic growth stage were selected, harvested from the culture dish with a cell scraper, and added into the medium containing 10% calf serum RPMI-1640 to make cell suspension. The cell concentration was adjusted to 25,000 cells/mL and inoculated into the 96-well culture plate with 200 µL cell suspension added to each well. After 24 h incubation at 37 °C with 5% CO_2_ and 95% air, the cells adhered to the wall, and the supernatant was aspirated. The blank control group was added with 200 µL of RPMI-1640 medium containing 0.1% DMSO, and the drug group was added with 200 µL of RPMI-1640 medium containing different concentrations of 5DN and its metabolites. After 24 h, the supernatant was aspirated, and 100 µL of medium solution containing 0.1 mg/mL MTT was added to each well for incubation in a 5% CO_2_ incubator at 37 °C. After 2 h, the supernatant was aspirated, and 100 µL DMSO was added to each well. The culture plate was gently shaken to dissolve completely. The absorbance value at 570 nm wavelength was measured with a plate reader (Elx800TM absorbance microplate reader, BioTek Instrument, Winooski, VT, USA) and repeated three times.

### 2.4. Nitric Oxide Assay

The nitrite concentration in the culture media was measured as an indicator of NO production by the Griess reaction [22]. RAW 264.7 cells at the logarithmic growth stage were selected, harvested from the culture dish with a cell scraper, and added into the medium containing 10% calf serum RPMI-1640 to make cell suspension. The cell concentration was adjusted to 25,000 cells/mL and inoculated into the 96-well culture plate with 200 µL cell suspension added to each well. After 24 h incubation at 37 °C with 5% CO_2_ and 95% air, the cells adhered to the wall, and the supernatant was aspirated. The blank control group was added with 200 µL of RPMI-1640 medium containing 0.1% DMSO, and the drug group was added with 200 µL of RPMI-1640 medium containing different concentrations of 5DN and its metabolites. After 24 h, 100 µL of supernatant was added to another 96-well culture plate from each well, and then 100 µL of the same volume of Griess reagent (A:B = 1:1) was added to each well. The plate was gently shaken and mixed and incubated in the incubator at 37 °C and 5% CO_2_. After 10 min, the absorbance value at 540 nm wavelength was measured with a plate reader and repeated 3 times. The concentration of NO was calculated according to the NaNO_2_ standard curve.

### 2.5. ELISA for Interleukin-1β (IL-1β)

RAW 264.7 cells (5 × 10^6^ cells/well) were seeded in 6-well plates. After 24 h, cells were treated with 1 µg/mL LPS alone or with serial concentrations of 5DN and its metabolites in 2 mL of serum complete media. After 24 h incubation, the culture media were collected and analyzed for IL-1β (R&D Systems, Minneapolis, MN, USA) level by ELISA kits, according to the manufacturer’s instructions.

### 2.6. Preparation of Whole-Cell Lysate

Whole-cell protein was collected as we described previously [23,24]. RAW 264.7 cells were first washed with ice-cold PBS and collected with a cell scraper from culture plates. The cells were combined with floating cells, if any, and incubated on ice in lysis buffer containing 20 mM Tris-HCl (pH 7.5), 150 mM NaCl, 1 mM EGTA, 1 mM EDTA, 1% Triton, 2.5 mM sodium pyrophosphate, 1 mM β-glycerophosphate, 1 mM Na_3_VO_4_, and 1 µg/mL leupeptin with freshly added protease inhibitor cocktail (Protease Inhibitor Cocktail Set III; Boston Bioproducts, Milford, MA, USA) for 20 min on ice. Cell suspensions were then subjected to sonication (5 s, three times). After further incubation for 20 min on ice, supernatants were collected by centrifugation at 10,000× *g* for 10 min. Protein concentrations were determined by a BCA protein assay kit (Pierce Biotechnology, Rockford, IL, USA), following the manufacturer’s instructions.

### 2.7. Immunoblot Analysis

Immunoblotting was performed as we previously described [23,24]. SDS-PAGE gel (8%) was placed in an electrophoresis tank, and a denatured protein sample (50 µg) and protein standard were sampled into the sample addition hole. The gel was run at a constant 150 volts until the protein standard band was completely separated and bromophenol blue reached the adhesive base (approx 1 h). Then the gel was transferred to nitrocellulose membranes for 2 h. The blot containing the transferred protein was blocked in blocking buffer (5% nonfat dry milk, 1% Tween-20 in 20 mM Tris-buffered saline, pH 7.6) for 2 h at room temperature, then incubated with the primary antibody in blocking buffer overnight at 4 °C followed by incubation with horseradish peroxidase-conjugated anti-mouse or anti-rabbit secondary antibody for 2 h, and finally detected by chemiluminescence (Boston Bioproducts) and autoradiography using Hyperfilm obtained from Kodak BioMax (Rochester, NY, USA). Antibodies for iNOS, COX-2, and HO-1 were purchased from Cell Signaling Technology (Danvers, MA, USA). Anti-β actin antibody was from Sigma-Aldrich (St. Louis, MO, USA).

### 2.8. Quantitative Real-Time Reverse-Transcription Polymerase Chain Reaction (qRT-PCR) Analysis

The total RNA of RAW 264.7 cells was extracted using RNeasy Plus Mini Kit (QIAGEN, Germantown, MD, USA) according to the manufacturer’s instructions. The RNA samples were detected by 1% agarose gel electrophoresis containing ethidium bromide, and the concentration and purity of RNA were determined by NanoDrop1000 spectrophotometer. From each sample, 0.16 mg RNA was extracted, and the reverse-transcription cDNA was synthesized and amplified by Brilliant II SYBR Green QRT-PCR Master Kit, 1-Step (Agilent, Santa Clara, CA, USA). The prepared reaction system was placed in the fluorescence real-time quantitative PCR instrument (Mx3000P QPCR system) for quantitative amplification and real-time detection. The primer pairs were synthesized by Integrated DNA Technologies, Inc. (Coralville, IA, USA) and the sequences were as followed: iNOS, forward primer 5′-TCC TAC ACC ACA CCA AAC -3′, reverse primer 5′-CTC CAA TCT CTG CCT ATC C -3′; COX-2, forward primer 5′-CCT CTG CGA TGC TCT TCC -3′, reverse primer 5′-TCA CAC TTA TAC TGG TCA AAT CC -3′ [25]; HO-1, forward primer 5′-AAG AGG CTA AGA CCG CCT TC -3′, reverse primer 5′-GTC GTC GTC AGT CAA CAT GG -3′; IL-1β, forward primer 5′-GAG TGT GGA TCC CAA GCA AT -3′, reverse primer 5′-CTC AGT GCA GGC TAT GGA CCA -3′ [26]; GAPDH, forward primer 5′-TCA ACG GCA CAG TCA AGG -3′, reverse primer 5′-ACT CCA CGA CAT ACT CAG C -3′ [25]. A minimum of three independent experiments were carried out, and each experiment had triplicate samples for each treatment. The copy number of each transcript was calculated relative to the GADPH copy number using the 2^−∆∆Ct^ method [27].

### 2.9. Statistical Analysis

All data were presented as mean ± SD. Student’s *t*-test was used to test the mean difference between the two groups. One-way analysis of variance (ANOVA) and Tukey’s post hoc test was used for the comparison of differences among more than two groups. Both 5% and 1% significant levels were used for the tests.

## 3. Results

### 3.1. 5DN and Its Metabolites (M1, M2, and M3) Inhibit NO Production in LPS-Induced RAW 264.7 Cells

**Figure 1:**
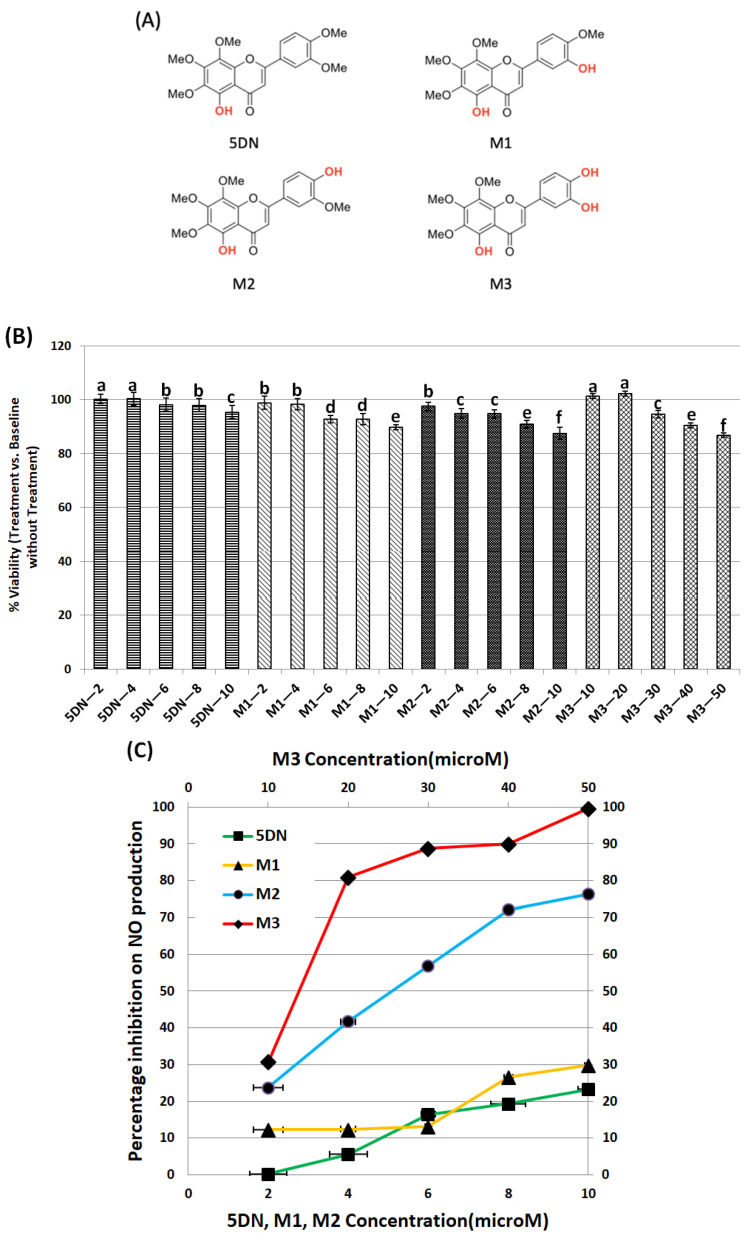
Inhibitory effects of 5DN and its metabolites M1, M2, and M3 on LPS-induced NO production within nontoxic dose ranges in RAW 264.7 cells. (**A**) The structures of 5DN and its metabolites M1, M2, and M3. (**B**) Cytotoxicity profiles of 5DN and its metabolites M1, M2, and M3 in RAW 264.7 macrophage cells. The viability of control cells was set as the reference with a value of 100%. Results are presented as mean ± SD (n = 6). Different superscript letters in each column indicate significant differences between groups (*p* < 0.05, n = 6) by one-way ANOVA and Tukey’s post hoc test. (**C**) Percentage of inhibition on NO production by 5DN and its metabolites M1, M2, and M3 in LPS-stimulated RAW 264.7 cells. The cells were treated with LPS (positive control) or LPS plus serial concentrations of 5DN and its metabolites M1, M2, and M3, as indicated in the bar graph. After 24 h of incubation, the culture media were collected for NO assay, as described in the Method section. Results are presented as mean ± SD. All treatments showed statistical significance in comparison with the positive control (*p* < 0.01, n = 6).

In this study, the nontoxic dose ranges of 5DN and its metabolites M1, M2, and M3 (Figure 1A) on RAW 264.7 cells were first determined. The effects of different concentrations of 5DN and its metabolites M1, M2, and M3 on the viability of RAW 264.7 cells were determined by MTT assay. The results showed that the viability of RAW 264.7 cells was not significantly decreased when the concentrations of 5DN and its metabolites M1 and M2 were lower than 10 µM (>90% viable cells compared to the control, Figure 1B). However, the maximum nontoxic concentration of metabolite M3 could reach 50 µM (Figure 1B). Thus, in the following assays, 5 and 10 µM of 5DN, M1, and M2, and 5, 10, and 20 µM of M3 would be used to ensure that any diminished inflammatory markers we observed were due to the anti-inflammatory effect of the test compounds rather than decreased cell numbers.

To investigate the anti-inflammatory property of 5DN and its metabolites, we tested their inhibitory effects on NO production in LPS-induced RAW 264.7 cells. As shown in Figure 1C, LPS surged the production of NO compared to the blank control group. When cells were treated with 5DN or its metabolites M1, M2 (all at 2–10 µM), and LPS for 24 h, M2 significantly reduced NO content by 24–76%. However, 5DN and M1 only reduced NO content by 23 and 30%, respectively, at the maximum concentration of 10 µM. Compared with the LPS group, M3 significantly inhibited NO production at 10–50 µM, with an inhibition rate of 31–100%. Notably, the inhibitory effect of M2 at 10 µM (NO inhibition rate: 76%) was stronger than that of M3 at the same concentration (NO inhibition rate: 31%).

### 3.2. 5DN and Its Metabolites (M2 and M3) Suppress iNOS and COX-2 Gene Expression in LPS-Induced RAW 264.7 Cells

**Figure 2:**
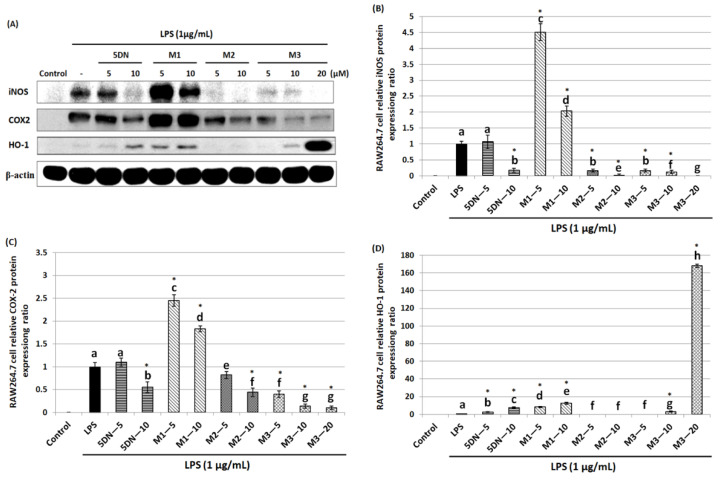
Effects of 5DN and its metabolites M1, M2, and M3 on the protein levels of iNOS (**A**,**B**), COX-2 (**A**,**C**), and HO-1 (**A**,**D**) in LPS-stimulated RAW 264.7 macrophages. The numbers underneath the blots represent band intensity (normalized to β-actin, means of three independent experiments) measured by Image J software. The standard deviations (all within ± 15% of the means) were not shown. β-actin was served as an equal loading control. Different superscript letters in each column indicate significant differences between groups (*p* < 0.05, n = 3) by one-way ANOVA and Tukey’s post hoc test. “*” indicates statistical significance compared to the control (*p* < 0.05, n = 3).

Since 5DN and its metabolites M1, M2, and M3 inhibited LPS-induced production of NO in RAW 264.7 cells, the effects of these compounds on the iNOS protein level were next examined. iNOS protein expression was detected by Western blot after 24 h treatment of RAW 264.7 cells with LPS and different concentrations of 5DN or its metabolites. The results showed that compared with LPS-positive control group, 5DN, M2, and M3 could significantly reduce the expression level of iNOS protein, while M1 had the opposite effect (Figure 2A,B). M3 showed the strongest inhibitory effect; for example, it could completely inhibit the expression of iNOS protein at 20 µM. It should be noted that the inhibitory effect of M2 on iNOS protein expression at 10 µM (inhibition rate: 98%) was stronger than that of M3 at the same concentration (inhibition rate: 88%). In addition, the effects of these compounds on COX-2 protein expression were also measured. Similar to the changes in iNOS protein level, 5DN, M2, and M3 could significantly reduce the expression level of COX-2 protein, while M1 also had an opposing effect (Figure 2A,C). The strongest inhibitory effect of M3 was observed at 10 µM, with an inhibition rate of 90%. These results indicated that 5DN and its metabolites M2 and M3 could inhibit LPS-induced iNOS and COX-2 expression at the transcriptional level.

**Figure 3:**
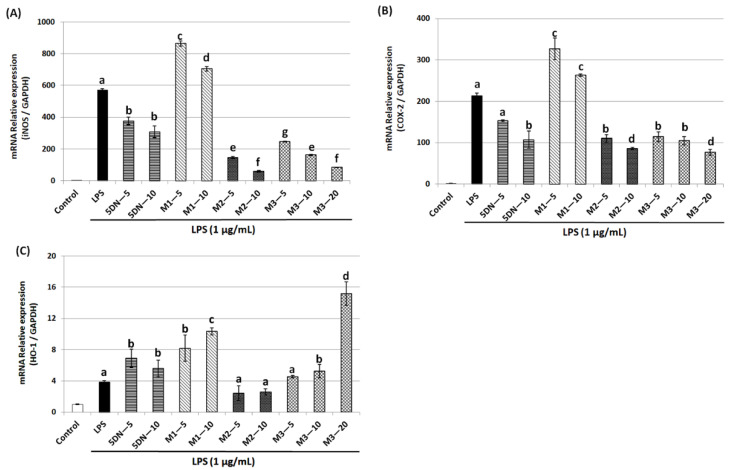
Effects of 5DN and its metabolites M1, M2, and M3 on the mRNA levels of iNOS (**A**), COX-2 (**B**), and HO-1 (**C**) in LPS-stimulated RAW 264.7 macrophages. Relative iNOS, COX-2, and HO-1 mRNA expression (2^−∆∆Ct^) was determined by real-time PCR and calculated by the Ct value for iNOS, COX-2, and HO-1 from GAPDH mRNA. ∆∆Ct = (C_t target gene_-C_t GAPDH_)-C_t control_-C_t GAPDH_). Each value represents the mean ± SD. Different superscript letters in each column indicate significant differences between groups (*p* < 0.05, n = 3) by one-way ANOVA and Tukey’s post hoc test.

Using the qRT-PCR technique, we further investigated whether 5DN and its metabolites suppressed LPS-stimulated induction of iNOS and COX-2 via a pre-translational mechanism. As shown in Figure 3A,B, LPS significantly induced the mRNA expression of iNOS and COX-2, which was consistent with the changes in their protein levels in response to LPS. 5DN, M2, and M3 could significantly reduce the mRNA level of iNOS and COX-2, while M1 again showed a reverse trend. The inhibition rate of M2 on iNOS mRNA level at 10 µM (inhibition rate: 90%) was greater than that of M3 at 20 µM (inhibition rate: 85%). Altogether, these observations suggest that 5DN, M2, and M3 could inhibit the expression of iNOS and COX-2 at the transcription levels.

### 3.3. 5DN and Its Metabolites (M2 and M3) Attenuate IL-1β Gene Expression in LPS-Induced RAW 264.7 Cells

**Figure 4:**
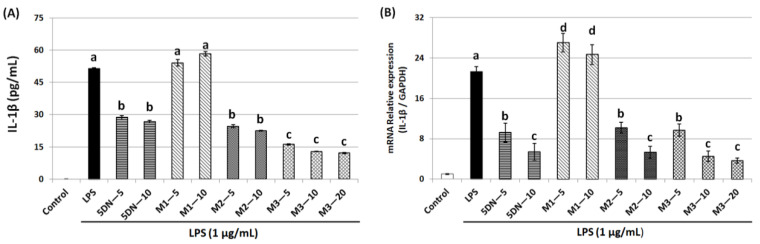
Inhibitory effects of 5DN and its metabolites M1, M2, and M3 on LPS-induced IL-1β expression in RAW 264.7 cells at protein (**A**) and mRNA (**B**) levels. Results are presented as mean ± SD. Different superscript letters in each column indicate significant differences between groups (*p* < 0.05, n = 3) by one-way ANOVA and Tukey’s post hoc test.

The pro-inflammatory factor IL-1β plays an important role in the development of many diseases, including cancer, so this study examined the effect of 5DN and its metabolites (M1, M2, and M3) on LPS-induced IL-1β production in RAW 264.7 cells. ELISA results showed that LPS drastically elevated IL-1β production at both protein and mRNA levels (Figure 4A,B). Compared with the LPS-positive control group, 5DN, M2, and M3 significantly reduced the protein expression level of IL-1β, while M1 caused no effect on IL-1β protein and a slight increase in IL-1β mRNA. M3 had the strongest inhibitory effect on the expression of IL-1β protein. Specifically, 5 µM of M3 reduced the content of IL-1β protein by 69%, which was stronger than that of other test compounds at 10 µM.

### 3.4. 5DN and Its Metabolites (M1 and M3) Increase HO-1 Gene Expression in LPS-Induced RAW 264.7 Cells

HO-1 is a phase II antioxidant enzyme that plays an important role in regulating inflammation [28]. Therefore, this study further examined the effects of 5DN and its metabolites (M1, M2, and M3) on the expression level of HO-1 in LPS-induced RAW 264.7 cells. Western blotting results showed that HO-1 protein expression levels were increased by 7.72-, 12.38-, and 3.04-fold when 5DN, M1, and M3 were at 10 µM, respectively, compared with the LPS control group (Figure 2A,D). When the concentration of M3 increased to 20 µM, the protein expression of HO-1 increased drastically to 168.17-fold. Then, the effects of 5DN and its metabolites (M1, M2, and M3) on the mRNA level of HO-1 were further analyzed. The results of qRT-PCR showed that 5DN, M1, and M3 could induce the mRNA expression of HO-1, while M2 had no effect (Figure 3C), which was similar to the results observed at the protein level.

## 4. Discussion

Studies have shown that the metabolites of bioactive substances are usually closely related to the biological characteristics, practical applications, and use limitations of their parent compounds [29]. Therefore, it is of great significance to study these metabolites. In this study, we investigated the inhibitory effects of 5DN and its in vivo metabolites M1, M2, and M3 on LPS-induced inflammation in RAW 264.7 cells. Our results, for the first time, showed that 5DN and its three metabolites had inhibitory effects on LPS-induced NO production in RAW 264.7 cells (Figure 1C). None of the experimental concentrations had toxic effects on RAW 264.7 cells, ensuring that the compound’s inhibition of NO was due to its anti-inflammatory effect and not to disruption of normal cell function. It is worth noting that M3 had a wider range of nontoxic doses than other compounds; for example, the maximum nontoxic concentration of M3 was 50 µM, while 5DN, M1, and M2 were only 10 µM (Figure 1B).

In order to understand the anti-inflammatory mechanisms of 5DN and its metabolites M1, M2, and M3, two key pro-inflammatory proteins, iNOS and COX-2, were detected by Western blot and qRT-PCR. The results showed that when the concentrations of 5DN, M2, and M3 were at 5 and 10 µM, the expression of iNOS and COX-2 protein was significantly inhibited in a dose-dependent manner (Figure 2A,C). The inhibitory effects of 5DN, M2, and M3 on iNOS and COX-2 protein expression were consistent with their inhibitory effects on total nitrite in culture media (Figure 1C). The results of qRT-PCR showed that 5DN, M2, and M3 could inhibit the expression of iNOS and COX-2 at the transcription level (Figure 3A,B). More importantly, when 5DN and M3 were used at the same concentrations (5 and 10 µM), not only the inhibitory rate of NO, but also the protein and mRNA levels of iNOS and COX-2 showed that M3 had a better anti-inflammatory effect than 5DN. However, M1 enhanced both the protein and mRNA levels of iNOS and COX-2, suggesting that M1 might inhibit the production of NO through other mechanisms.

The pro-inflammatory factor IL-1β can activate NF-κB binding κB element in NOS promoter to promote iNOS transcription [30], which also induces COX-2 expression [31]. High concentrations of pro-inflammatory factors can also stimulate tumor growth and development [32]. Compared with the LPS-positive control group, 5DN, M2, and M3 significantly attenuated the protein content of IL-1β, while M1 had no significant inhibitory effect (Figure 4A). 5DN, M2, and M3 were also found to suppress IL-1β mRNA levels (Figure 4B), suggesting that they inhibited IL-1β protein expression by interfering with its transcription. Because IL-1β can induce the expression of iNOS and COX-2, the inhibitory effect of 5DN, M2, and M3 on iNOS and COX-2 may be related to their inhibitory effects on IL-1β.

HO-1 is an important phase II antioxidant enzyme [28]. Studies have shown that HO-1 can inhibit inflammation and antagonize the pro-inflammatory effects of toxic substances [33,34,35,36,37,38,39]. In order to further understand the anti-inflammatory mechanisms of 5DN and its metabolites M1, M2, and M3, their effects on HO-1 were examined. The results showed that the induction of HO-1 might contribute to 5DN, M1, and M3′s anti-inflammatory effects, whereas M2 did not significantly alter HO-1 expression (Figure 2A,D and Figure 3C). With increasing concentration of M3, HO-1 protein expression even dramatically increased to 168.17-fold. Studies have shown that HO-1 can enhance the antioxidant capacity of cells by producing antioxidants, such as bilirubin, which can inhibit the expression of iNOS protein and the production of NO in RAW 264.7 cells [40,41]. Another major product of HO-1 activity is carbon monoxide (CO), which can inhibit COX-2 protein expression by inhibiting CCAAT enhancer binding protein (C/EBP) [42]. Studies have shown that CO can also inhibit the activity of the iNOS enzyme, thus reducing the production of NO [43]. These results may explain why M1 reduced the content of NO in cells but exerted no inhibition on iNOS or COX-2.

We found that metabolites M2 and M3 had stronger anti-inflammatory activities than their parent compound, 5DN, which is related to their structural properties. In the structure of 5DN, the 3’-site methoxy in the B ring is hydroxylated, which is M1. In the structure of 5DN, the 4’-site of B-ring methoxy is hydroxylated, which is M2. M3 is hydroxylated at the 3’- and 4’- sites of the B ring in the 5DN structure. The phenol hydroxyl group and the charged amino acid residues can form ionic bonds, and the protein conformation may be affected, resulting in a change in protein activity [44]. It has been reported that the hydroxyl groups of flavonoids could affect their inhibitory effects on the expression of iNOS and COX-2 [45]. In addition, the anti-inflammatory efficacy of metabolites M1, M2, and M3 on LPS-indued inflammation in RAW 264.7 cells were in the order of M3 > M2 > M1, suggesting that different positions and quantities of hydroxyl groups in the B-ring impact their binding to signaling proteins (e.g., different binding sites or the same site but with different binding forces), which ultimately leads to different biological properties.

In summary, 5DN and its in vivo metabolites M1, M2, and M3 inhibited LPS-induced inflammation in RAW 264.7 cells. The anti-inflammatory activities of 5DN, M2, and M3 were mainly achieved by inhibiting the expression of iNOS, COX-2, and IL-1 β genes. The anti-inflammatory activity of M1 was related to its induction of HO-1 gene expression. To our knowledge, this is the first systematic comparative study of the anti-inflammatory properties of 5DN and its metabolites. More importantly, the results showed that metabolites M2 and M3 generally had stronger anti-inflammatory efficacy than 5DN. The relationship between their structural properties and anti-inflammatory activities is worthy of further investigation. In addition, more inflammatory signaling pathways, such as TLR4, NF-κB, and IκBα, should be analyzed in future studies to understand the anti-inflammatory mechanisms of 5DN and its metabolites more comprehensively. Furthermore, knockdown/knockout and in vivo studies would be useful to achieve a better understanding of the biological activities of 5DN and its metabolites.

## Data Availability

Raw data obtained in this study are available from the corresponding authors upon reasonable request.
